# Supplementary material for: Complexome profiling of the Chlamydomonas psb28 mutant reveals TEF5 as an early PSII assembly factor
Source: Plant Cell. 2025 Mar 17;37(6):koaf055. doi: 10.1093/plcell/koaf055 (PMC12164590; doi:10.1093/plcell/koaf055)
Supplement: koaf055_Supplementary_Data [file koaf055_supplementary_data.zip › psb28_interactive.zip!/plots/intensity/qualityPlots/peptides/SRP43_Cre04.g231026.t1.1.html]

### Cre04.g231026.t1.1

Trivial name: SRP43  
SeqCoverage [median]: 24.00  
Mass: 46.20 kDa  
  
 

| Sequence | MissedCleavage | Mass [Da] | wtIntensity | muIntensity | Score |
| --- | --- | --- | --- | --- | --- |
| AGPPALVYAEGYKEVEELAGAR | 1 | 2289.16 | 6646.72 | 0.00 | 115.76 |
| ASMPLSMGSVQR | 0 | 1262.61 | 14445.76 | 6927.21 | 152.17 |
| DNTGRDVVNLIENLR | 1 | 1726.90 | 1952.22 | 0.00 | 68.04 |
| DVVNLIENLR | 0 | 1183.66 | 6503.65 | 0.00 | 142.98 |
| EFLVSFGDGRDDEWVPER | 1 | 2151.99 | 1650.12 | 0.00 | 88.73 |
| ELLAHVVDENRR | 1 | 1449.77 | 7414.87 | 5780.37 | 84.48 |
| EVEELAGAR | 0 | 972.49 | 336.92 | 0.00 | 66.07 |
| KADVDAMIK | 1 | 989.52 | 5180.67 | 1314.71 | 80.23 |
| VVVESDPPR | 0 | 996.52 | 267.02 | 0.00 | 55.53 |
| VVVESDPPRVEYLVK | 1 | 1727.95 | 14019.78 | 16218.59 | 88.09 |
|  |  |  | 58417.7 | 30240.9 |  |
